# Maternal serum unmetabolized folic acid concentration following multivitamin and mineral supplementation with or without folic acid after 12 weeks gestation: A randomized controlled trial

**DOI:** 10.1111/mcn.13668

**Published:** 2024-05-23

**Authors:** Dian C. Sulistyoningrum, Thomas R. Sullivan, Monika Skubisz, Debra J. Palmer, Simon Wood, Per Magne Ueland, Adrian McCann, Maria Makrides, Timothy J. Green, Karen P. Best

**Affiliations:** ^1^ SAHMRI Women and Kids Theme South Australian Health and Medical Research Institute Adelaide South Australia Australia; ^2^ Faculty of Health and Medical Sciences The University of Adelaide Adelaide South Australia Australia; ^3^ School of Public Health, Faculty of Health and Medical Sciences The University of Adelaide South Australia Australia; ^4^ Telethon Kids Institute University of Western Australia Nedlands Western Australia Australia; ^5^ School of Medicine University of Western Australia Crawley Western Australia Australia; ^6^ School of Public Health, Faculty of Health Sciences Curtin University Perth Western Australia Australia; ^7^ Food, Nutrition and Health Program University of British Columbia Vancouver British Columbia Canada; ^8^ InovoBiologic Inc. Calgary Alberta Canada; ^9^ Bevital AS, Laboratoriebygget Bergen Norway; ^10^ College of Nursing and Health Sciences Flinders University Bedford Park South Australia Australia

**Keywords:** folic acid, periconception, pregnancy, prenatal supplementation, red blood cell folate, unmetabolized folic acid

## Abstract

Pregnant women are advised to take folic acid (FA) supplements before conception and during the first trimester of pregnancy. Many women continue FA supplementation throughout pregnancy, and concerns have been raised about associations between excessive FA intake and adverse maternal and child health outcomes. Unmetabolized folic acid (UMFA) is found in serum after high FA intakes and is proposed as a biomarker for excessive FA intake. We aimed to determine if removing FA from prenatal micronutrient supplements after 12 weeks of pregnancy reduces serum UMFA concentrations at 36 weeks gestation. In this double‐blind, randomized controlled trial conducted in South Australia, 103 women with a singleton pregnancy were randomly assigned at 12–16 weeks gestation to take a micronutrient supplement containing no FA or 800 µg/day FA from enrollment until 36 weeks gestation. Ninety women (0 µg/day FA *n* = 46; 800 µg/day FA *n* = 44) completed the study. Mean, UMFA concentration was lower in the women randomized to the 0 µg/day group compared to the 800 µg/day FA group, 0.6 ± 0.7 and 1.4 ± 2.7 nmol/L, respectively. The adjusted mean difference (95% CI) in UMFA between the groups was [‐0.85 (−1.62, −0.08) nmol/L, *p* = 0.03]. Maternal serum and red blood cell folate concentrations were lower in the 0 µg/day FA group than in the 800 µg/day group (median 23.2 vs. 49.3 and 1335 vs. 1914 nmol/L, respectively; *p* < 0.001). Removing FA at 12–16 weeks gestation from prenatal micronutrient supplements reduced the concentration of UMFA at 36 weeks gestation.

## Key messages

- Folic acid (FA) supplementation is needed before pregnancy and during the first trimester to reduce neural tube defects.
- After the first trimester, FA has no proven benefit, but many women continue FA supplementation throughout pregnancy.
- There are concerns excessive FA supplementation later in pregnancy may increase risk of adverse pregnancy and child outcomes. Unmetabolized FA is a biomarker of excessive FA intakes.
- We have shown that discontinuing FA supplementation after 12 weeks of gestation results in lower maternal unmetabolized FA concentrations.
- Larger studies are required to determine how continuing FA beyond the first trimester affects maternal and child health outcomes.

## Graphical abstract

Unmetabolized folic acid (FA) is a biomarker of excessive FA intake. Removing FA from prenatal supplements after the first trimester reduces maternal serum unmetabolized FA levels by 36 weeks of pregnancy.

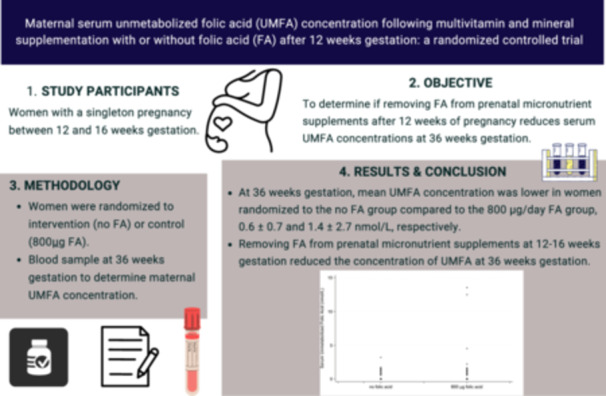

## 1 INTRODUCTION

Neural tube defects (NTDs) are birth defects caused by the failure of the neural tube to close properly, which occurs ~28 days postconception (Botto et al., 1999). Folic acid (FA) taken before conception and during early pregnancy reduces a woman's risk of having an NTD‐affected pregnancy (Berry et al., 1999; Czeizel & Dudás, 1992; MRC Vitamin Study Research Group, 1991). In response, health authorities in many countries recommend women take an FA‐containing supplement before conception (Royal Australian and New Zealand College of Obstetricians and Gynaecologists, 2016; World Health Organization, 2016). In Australia, women are advised to take a supplement containing 500 µg of FA daily for at least 1 month before trying to conceive and for the first 3 months of pregnancy (Royal Australian and New Zealand College of Obstetricians and Gynaecologists, 2016). Although there is no conclusive evidence for any overall benefit of FA supplementation beyond 12 weeks gestation (31 trials involving 17,771 women) (De‐Regil et al., 2015), many women continue to take FA supplements throughout their whole pregnancy, typically at amounts up to 800 µg/day or higher (Shand et al., 2016). In addition, as NTDs occur in the first month of pregnancy and many pregnancies are unplanned, more than 80 countries, including Australia, Canada and the USA, have mandated fortification of staple foods with FA, further increasing FA intakes of pregnant women (Murphy & Westmark, 2020).

The common practice of continuing FA supplementation beyond the first trimester, especially in countries with staple foods FA fortification, is concerning due to increasing reports suggesting excessive FA intakes in late pregnancy may be associated with adverse maternal and child health outcomes, including an increased risk of gestational diabetes (Karaçil Ermumcu & Acar Tek, 2023; Kintaka et al., 2020; Li et al., 2019), allergic disease (McGowan et al., 2020; Ogawa et al., 2018; Roy et al., 2018), and obesity and metabolic dysfunction in the child later on (Yajnik et al., 2008). Although findings from observational studies have been inconsistent, evidence from randomized controlled trials (RCTs) is lacking. The suggestion of risk necessitates further exploration of excessive FA intake beyond the first trimester.

FA is a synthetic form of folate not found naturally in food. Because of its high bioavailability and stability, it is the form of folate used in supplements and to fortify food (Greenberg et al., 2011). When consumed, FA is reduced and methylated to 5‐methyltetrahydrofolate (5‐MTHF) in the enterocyte or liver. At higher intakes, the enzymes required to convert FA to 5‐MTHF are saturated, and the excess FA circulates in its unmetabolized form (UMFA) (Kelly et al., 1997). UMFA has been proposed as a potential biomarker of excessive FA intake (Kelly et al., 1997). Concerns have been raised over whether high concentrations of circulating UMFA may adversely affect the developing fetus (Smith et al., 2008). In acute dosing studies in nonpregnant individuals, UMFA rises rapidly after FA ingestion and falls over the following hours (Kelly et al., 1997; Sweeney et al., 2007). The greater the dose of FA, the higher the UMFA concentration and the longer it is detected in serum. The effect of chronic excessive intake of FA on UMFA concentrations is less clear.

Unmetabolized FA has been detected in maternal blood samples in several population studies (Best et al., 2020; Obeid et al., 2010; Plumptre et al., 2015; West et al., 2012) and one RCT in a country without mandatory fortification (Pentieva et al., 2016). However, there are no published RCTs investigating the effect of prolonged intake of commonly used higher‐dose prenatal FA‐containing supplements combined with background intakes from mandatory fortification of staple foods on UMFA concentration.

Without a doubt, it is crucial to take FA supplements in early pregnancy to reduce NTDs. However, supplementation beyond this time is in question. We aimed to investigate the effect of removing FA from prenatal supplements after 12 weeks gestation compared with the common practice of continuing FA supplementation of 800 µg/day throughout pregnancy on maternal serum UMFA at 36 weeks gestation.

## 2 METHODS

This trial was a multicenter, double‐blind, placebo‐controlled, parallel‐group (1:1 allocation ratio) RCT. The trial protocol, published previously (Sulistyoningrum et al., 2020) was developed by the authors and approved by the Women's and Children's Health Network Research Ethics Committee—HREC/19/WCHN/018 and Flinders Medical Centre—SSA/20/SAC/61. The trial was conducted according to the 2007 National Statement on Ethical Conduct in Human Research and the Note for Guidance on Good Clinical Practice (CPMP/ICH/135/95) and prospectively registered with the Australia New Zealand Clinical Trials Registry—ACTRN12619001511123.

### 2.1 Study participants and setting

Pregnant women living in South Australia were recruited to the trial between December 2019 and November 2020. Women with a singleton pregnancy between 12^+0^ and 16^+0^ weeks gestation who were taking a FA‐containing supplement and planned to continue it throughout pregnancy were eligible to participate. Women were excluded if they were carrying a fetus with a confirmed or suspected fetal abnormality, had a prior history of an NTD‐affected pregnancy or were taking medications that interfere with folate metabolism. Women were recruited in person at their first antenatal clinic appointment or remotely through a Trial Recruitment Company (TrialFacts Australia), which utilizes an online digital marketing campaign and an electronic pre‐screening survey.

### 2.2 Randomization, blinding and masking

After obtaining written informed consent, women were randomized by research personnel using a secure web‐based randomization service and stratified by gestational age at trial entry 12^+0^ to ≤14^+0^ weeks or >14^+0^ to 16^+0^ weeks gestation. Allocation followed a computer‐generated randomization schedule using randomly permuted blocks of sizes 4 and 6 (1:1 ratio), prepared by an independent statistician not involved with trial participants or data analysis. A unique and uninformative four‐digit study identification number (Study ID) was assigned to each participant. The intervention and control supplements were identical in size, shape, color, packaging and labeling and identified by a colored label only. Four colors were used to optimize blinding to group assignments (blue, pink, yellow and green). Color matching to the unique study ID was prepared by an independent statistician not involved with trial participants or data analysis. Participants, researchers and laboratory personnel remained blinded to the group assignments until the data analysis was complete.

### 2.3 Trial interventions

Women in the intervention group received multivitamins and mineral supplements without FA (0 µg FA/day). Women in the control group were assigned the same formulation with 800 µg of FA/day (Supporting Information: Table 1), as standard practice is to take a prenatal supplement containing FA and the most common brand of supplements used in Australia contains this dose. Following randomization, women were given two bottles containing 125 caplets and advised to cease any other FA‐containing supplements for the duration of the trial. The assigned study supplements were taken once daily from trial entry (12–16 weeks gestation) until the day before the clinic visit and blood draw at 36 weeks gestation. Intervention and control supplements (PreNuro®) were formulated to provide daily multivitamin and mineral levels for prenatal supplementation. They were manufactured in a licensed facility following the Code of Good Manufacturing Practice of Medicinal Products (Therapeutic Goods Administration, 2018) by The Factors Group of Nutritional Companies Inc. The company had no other role in the trial.

### 2.4 Data collection

Baseline characteristics were collected at enrollment and included gestational age, maternal age, height and weight, race, education, pre‐pregnancy and current supplement use, annual household income, parity, and alcohol intake and smoking in the 3 months leading up to pregnancy. Women were asked to complete an electronic 80‐item food frequency questionnaire (FFQ) (The Dietary Questionnaire for Epidemiological Studies v3.2, Cancer Council, Victoria) at enrollment (baseline) and 34 weeks gestation to estimate folate intakes from foods. Adherence to the trial regimen and the occurrence of any adverse events were assessed by monthly electronic surveys sent by short message surveys or phone calls by study staff. Women returned for an in‐person visit at 36 weeks gestation so that the number of unused caplets could be recorded and trained research personnel could obtain a venous blood sample. Women were asked to refrain from taking their study supplements and consuming foods high in FA on the day of sample collection. Birth data, including gestational age, weight, length and head circumference, were extracted from maternal and infant medical records or parental reports.

### 2.5 Blood sample collection

A 10 mL non‐fasting venous blood sample was collected into two evacuated containers containing no anticoagulant and ethylenediaminetetraacetic acid (EDTA) (BD Vacutainer®). The EDTA vacutainer was inverted 10 times, and an aliquot was placed in a cryovial, diluted 1 in 11 with 1% ascorbic acid and incubated for 30 min at 37°C. The serum vacutainer was left to clot at room temperature for at least 30 min. Vacutainers were centrifuged at 1500 *g* for 15 min at 4°C, and serum and plasma were aliquoted into cryovials and stored at ^‐^80°C until analyzed.

### 2.6 Blood analysis

A complete blood count was performed using an automated hematology analyzer by SA Pathology. Serum UMFA was measured using the liquid chromatography–tandem mass spectrometry‐based method of Hannisdal et al. (2009) at Bevital (www.bevital.no). The limit of detection (LOD) for serum UMFA was 0.27 nmol/L, and both within‐ and between‐day coefficient of variance (CV) was 7%. The method uses an isotope‐labeled FA internal standard, and a robotic workstation performs all sample processing.

Whole blood and serum folate concentrations were determined using the microbiological method, using standardized kits from the U.S. Centers for Disease Control and Prevention (US CDC) (US Centers for Disease Control and Prevention, 2018). This method is based on the technique of O'Broin and Kelleher (1992), uses 96‐well microplates, 5‐methyl tetrahydrofolate (Merck Eprova) as a calibrator, and chloramphenicol‐resistant *Lactobacillus rhamnosus* (ATCC 27773TM) as the test organism. High‐ and low‐quality controls (QC) provided by the US Centers for Disease Control and Prevention, whole blood and plasma folate, were run in quadruplets on every plate. RBC folate was calculated by subtracting plasma from whole blood folate and correcting for hematocrit. As per instructions (US Centers for Disease Control and Prevention, 2018), if all QC results were within mean (2 SD) limits, the assay was accepted; if more than one of the QC results were outside of the mean (2 SD) limits or any of the QC results were outside of the mean (3 SD) limits, then the assay was rejected. Results from assay runs that passed QC were used when the quadruplets were below 15%. If the quadruplets’ coefficient of variation (CV) was above 15%, the largest outlier was removed. The results were recorded if the CV of the remaining triplicates was below 10%; otherwise, the sample measurement was repeated.

At the population level, WHO recommends RBC folate concentrations be >906 nmol/L in women of reproductive age to prevent NTDs. This RBC folate value was generated using FA as the calibrator (Daly, 1995). We used a newer method recommended by the US CDC that uses 5‐methyl tetrahydrofolate as the calibrator. Since 5‐methyl tetrahydrofolate gives lower RBC folate concentrations than FA, we used a cutoff of >748 nmol/L to define the optimal RBC folate concentration for NTD risk reduction (Zhang et al., 2018).

### 2.7 Outcome measures

The primary outcome was the concentration of UMFA in maternal serum at 36 weeks gestation. Secondary outcomes included maternal serum and RBC folate concentrations at 36 weeks gestation and birth outcomes, including gestational age, birth weight, length and head circumference.

### 2.8 Changes to outcomes and trial design

We adapted some aspects of our methodology due to the COVID‐19 pandemic. As per the CONSERVE statement (Orkin et al., 2021), we have described our original methods (Sulistyoningrum et al., 2020) and our adaptations as follows (Gould et al., 2021). When the trial commenced in December 2019, women were recruited from antenatal clinics, and a baseline blood sample was collected at enrollment. In March 2020, due to COVID‐19 restrictions in South Australia, in‐person enrollment was suspended, and we could no longer collect the baseline blood sample. Eighteen women were recruited before in‐person enrollment was suspended. Screening methods were modified to include online screening, a digital marketing campaign and e‐consent using Research Electronic Data Capture (REDCap, Vanderbilt University). REDCap is a secure web application for building and managing online surveys and databases. Enrollment and all study visits up to 36 weeks gestation were conducted via telephone, and supplements were couriered to participants. Birth data could no longer be extracted from medical records and were obtained by maternal reports. Maternal and infant birth characteristics, such as gestational age, weight, length and head circumference, were collected to compare treatment groups, as this study was not powered to evaluate clinical outcomes. We would caution about concluding these outcomes due to the small sample size and lack of control for multiple testing.

### 2.9 Sample size and statistical analysis

A target sample of 90 women (45 per group) was chosen to provide >90% power to detect a standardized difference in mean UMFA concentration at 36 weeks gestation between groups of 0.60 (two‐tailed alpha = 0.05, correlation between UMFA concentrations at baseline and 36 weeks gestation = 0.60) (Pentieva et al., 2016). Calculations were performed based on a standardized mean difference (mean difference divided by SD of the outcome at 36 weeks gestation) due to considerable variability in the literature in the reported SD for UMFA concentration in pregnancy (McGowan et al., 2020; Pentieva et al., 2016).

All analyses were undertaken on an intention‐to‐treat basis (i.e., participants were analyzed as randomized, irrespective of compliance). UMFA values were compared between groups using linear regression, with a robust variance estimator employed to allow for unequal variances between groups. Values below the detection limit were treated as a 0 in the analysis. Secondary outcomes were analyzed using linear regression models, with log transformations applied where appropriate to satisfy model assumptions better. All analyses were adjusted for gestational age at trial entry (12 to ≤14 weeks or >14 weeks) since this was used to stratify the randomization, with analyses of birth anthropometrics adjusted for infant sex. Analyses were based on participants with available data (complete case analysis), with estimation of the intention to treat effect proceeding under the assumption that outcome data were missing at random conditional on treatment group and covariates for adjustment. Statistical calculations were performed using Stata v18 (StataCorp LP).

## 3 RESULTS

### 3.1 Trial participants

**Figure 1:**
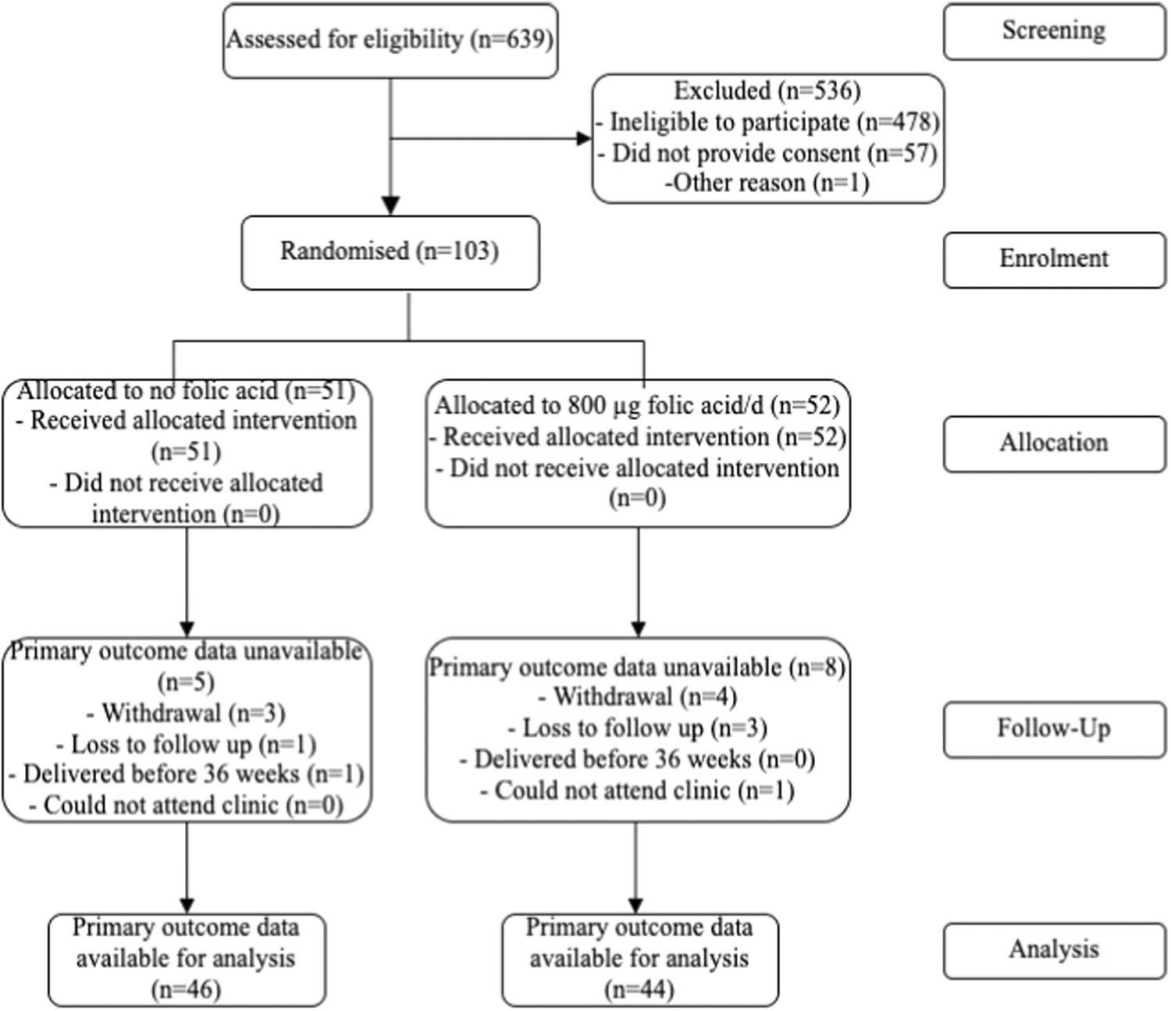
Participant flowchart.

A total of 103 women were randomized: 51 to the 0 µg FA/day supplement group (intervention) and 52 to the 800 µg FA/day supplement (control) group. After withdrawal of consent (*n* = 7), loss to follow‐up (*n* = 4), inability to attend the clinic visit (*n* = 1) and preterm birth before 36 weeks gestation (*n* = 1), primary outcome data were available for 90/103 (87%) of women (Figure 1). The average age of women entering the trial was 31 years, and more than 80% of the participants were Caucasian. Most women (87%) had completed secondary education, and 55% had an annual household income higher than AUD$105,000. Overall mean total folate intake (SD) was 585 (264) µg/day dietary folate equivalent (DFE) at baseline and 559 ± 253 µg/day DFE at 36 weeks (Table 1). Adherence to the trial supplements was similar between the intervention and control groups, with 85% of women who returned bottles consuming >80% of supplements to 36 weeks gestation. These results were comparable with results from compliance questioning at study visits. Similar percentages of women met the definition of high compliance in each group, 84% in the control group (37/52 returned bottles), compared to 85% in the no FA intervention group (39/51 returned bottles).

**Table 1:** Maternal baseline characteristics and folate intake at 36 weeks gestation.^a^

Characteristics	Intervention: no FA (*n* = 51)	Control 800 µg FA/day (*n* = 52)
Age, years	30.7 ± 5.2	31.4 ± 4.4
Gestational age at trial entry (weeks)		
12 to <14	32 (63)	32 (62)
≥14 to 16	19 (37)	20 (38)
Maternal BMI at enrollment (*n* = 94)	25.2 ± 5.0	27.0 ± 6.2
Ethnicity		
European	41 (80)	44 (85)
Other	10 (20)	8 (15)
Completed secondary education	46 (90)	44 (85)
Annual household income		
AUD$70,000 or less	9 (18)	9 (17)
AUD$70,001–$105,000	12 (24)	7 (13)
AUD$105,001–$205,000	23 (45)	26 (50)
>AUD$205,000	5 (10)	7 (13)
Prefer not to disclose	2 (4)	3 (6)
Parity		
0	27 (53)	20 (38)
Smoked tobacco in 3 months before pregnancy	5 (10)	5 (10)
Consumed alcohol in 3 months before pregnancy	34 (67)	41 (79)
Folate intake at baseline, µg/day (*n* = 88)		
Total dietary folate^b^	644 ± 298	528 ± 214
FA from fortified food	204 ± 127	155 ± 110
Natural food folate	303 ± 124	268 ± 88
Folate intake at 34 weeks, µg/day (*n* = 84)^c^		
Total dietary folate^b^	581 ± 269	538 ± 238
FA from fortified food	179 ± 131	152 ± 100
Natural food folate	282 ± 105	281 ± 103

^a^ Values are mean ± SD or *n* (%).

^b^ As dietary folate equivalents = 1.7 × µg FA from fortified food + µg natural food folate.

^c^ The *p* value for the mean difference in folate intake at 34 weeks between groups was 0.41 for total dietary folate, 0.29 for FA from fortified food and 0.98 for natural food folate.

### 3.2 Outcomes

**Figure 2:**
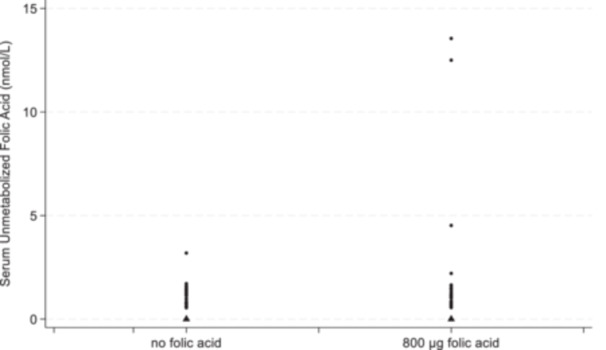
Unmetabolized folic acid by treatment group (triangles are for the values below the limit of detection).

At 36 weeks, mean UMFA was significantly lower in women in the 0 µg FA/day supplement group (0.6 ± 0.7 nmol/L) than in women who received 800 µg FA/day supplementation (1.4 ± 2.7 nmol/L, adjusted mean difference = −0.85 (95% CI, −1.62, −0.08) nmol/L, *p* = 0.03) (Figure 2). Maternal serum folate concentrations were lower in the 0 µg FA/day supplement group compared to the 800 µg FA/day supplementation group; median 23.2 versus 49.3 nmol/L, the ratio of geometric means 0.56 (95% CI, 0.46, −0.68 nmol/L), *p* < 0.001 (Table 2). Similarly, median RBC folate concentrations were significantly lower in the 0 µg FA/day supplement group than in the 800 µg FA/day supplementation group; 1340 versus 1910 nmol/L, the ratio of geometric means 0.69 (95% CI, 0.61–0.77), *p* < 0.001 (Table 2). Serum and RBC folate concentrations were within normal clinical range to indicate no folate deficiency in the intervention and control groups, >6.8 nmol/L for serum folate and >305 nmol/L for RBC folate concentrations (Supporting Information: Figure 1).

**Table 2:** Blood folate concentrations at 36 weeks and neonatal outcome by treatment group.

Outcome	Intervention no FA^a^	Control 800 µg FA/day^a^	Treatment effect^b^ (95% CI)	*p* Value
Serum unmetabolized FA (*n* = 90)^c^	0.6 ± 0.7	1.4 ± 2.7	−0.85 (−1.62, −0.08)	0.03
Serum folate, nmol/L (*n* = 90)	23.2 (18.0, 28.4)	49.3 (32.7, 57.7)	0.56 (0.46, 0.68)^d^	<0.001
Red blood cell folate, nmol/L (*n* = 90)	1340 (1150, 1510)	1910 (1530, 2300)	0.69 (0.61, 0.77)^d^	<0.001
Gestational age at birth, weeks (*n* = 86)	39.3 ± 1.7	39.0 ± 1.3	0.3 (−0.4, 1.0)	0.36
Birth weight, g (*n* = 90)	3331 ± 519	3383 ± 473	−44 (−255, 166)	0.68
Birth length, cm (*n* = 78)	49.3 ± 2.8	49.9 ± 2.7	−0.5 (−1.7, 0.8)	0.46
Birth head circumference, cm (*n* = 68)	34.1 ± 2.1	35.0 ± 1.3	−0.9 (−1.8, −0.1)	0.04

^a^ Values are median (IQR) or mean ± SD.

^b^ Adjusted for gestational age at trial entry for all outcomes and infant sex for birth anthropometric outcomes. Treatment effect expressed as a mean difference (95% CI) unless indicated otherwise.

^c^ Serum unmetabolized FA was detected in 50% of samples in the intervention group and 75% of samples in the control group.

^d^ Treatment effect expressed as a ratio of geometric means (95% CI).

### 3.3 Birth outcomes

There were no significant differences between the groups for birth gestational age, weight and length, except for head circumference, which was lower in the 0 µg FA/day supplement group compared to the 800 µg FA/day group (mean difference: −0.9 cm; 95% CI, −1.8, −0.1, *p* = 0.04) (Table 2).

### 3.4 Safety and adverse events

Adverse events were comparable between the groups, with nausea the most common symptom overall at 1 week post‐randomization (27%) and 20 weeks gestation (29%) (Supporting Information: Table 2). One infant in each group was admitted to the Neonatal Intensive Care Unit (classified as a serious adverse event). All serious adverse events were reviewed and categorized as unlikely to be related to the trial product or protocol.

## 4 DISCUSSION

We investigated the effect of removing FA from prenatal supplements after 12 weeks gestation on maternal UMFA concentrations in late pregnancy in a country with mandatory FA fortification of staple foods. UMFA concentrations were higher in those women randomized to the 800 µg FA/day supplement group compared to women in the 0 µg FA/day supplement group. UMFA concentrations were below the level of detection in only a quarter of women (11/44) in the 800 µg FA/day supplement group compared to half (23/46) of women in the 0 µg FA/day supplement group. Our results are dissimilar to the findings of the only other published RCT investigating the effect of prenatal FA supplementation on maternal UMFA concentration. Pentieva et al. (2016) reported that women randomized to FA supplements were more likely to have detectable plasma UMFA at 36 weeks gestation than women randomized to placebo (42% vs. 16%) but found no significant difference in the mean ± SD concentration of UMFA between groups (0.13 ± 0.49 vs. 0.44 ± 0.80, interaction *p*‐value = 0.38) (Pentieva et al., 2016). Our mean UMFA concentration was similar in the 0 µg/day FA‐supplemented group in the Pentieva et al. study but higher in those receiving 800 µg/day FA supplementation. The dose of FA used by Pentieva et al. (2016) was lower than that found in common prenatal multivitamin and mineral supplements in Australia and many other countries, which range from 500 µg to 1000 µg/day (Parr et al., 2017; Plumptre et al., 2015). Furthermore, the Pentieva et al. study was conducted in Northern Ireland, which only had voluntary (rather than mandatory) FA fortification of food (Pentieva et al., 2016). The prevalence of detectable UMFA in our trial participant population (62%) is lower than observational studies in pregnant women in Australia (93%, >0.03 to 244.7 nmol/L) (Best et al., 2020), USA (81%, 0.23–1.47 nmol/L) (West et al., 2012) and Canada (97%, 0.00–0.91 nmol/L) (Plumptre et al., 2015). However, UMFA concentrations differ substantially between studies and appear to be influenced by recent FA intakes (including ingestion of an FA containing supplement), which may explain the variability. Pfeiffer et al. reported detectable levels of UMFA in nearly all National Health and Nutrition Examination Survey (NHANES) participants (>95%, range >0.3–397 nmol/L) (Pfeiffer et al., 2015). NHANES is a representative sample of the US population, including men, women, and children. Although 38% of NHANES survey participants were fasting >8 h, Pfeiffer et al. reported that the detection of UMFA was evident regardless of fasting status, yet concentrations differed significantly by length of fasting (Pfeiffer et al., 2015).

We asked participants to avoid taking their study supplement on the day of their blood collection because we were interested in the long‐term effect of FA supplementation on UMFA, not the acute effect, as this is well established (Kelly et al., 1997; Sweeney et al., 2007; Zheng et al., 2015). Zheng et al. reported that following a single dose of 800 µg FA in 20 healthy male subjects, UMFA increased, peaking at around 2.5 h in plasma but returned to undetectable levels within 12 h (Zheng et al., 2015). Although we could detect UMFA in those receiving no FA from study supplements (our intervention group), we had expected that chronic dosing of FA from early pregnancy would result in substantially higher UMFA concentrations in the 800 µg/day FA supplementation group.

The serum and RBC folate differences were as expected and consistent with other prenatal FA supplementation trials (Crider et al., 2014; Obeid et al., 2018; Pentieva et al., 2016). At 36 weeks gestation, median serum folate was ~26 nmol/L lower and median RBC folate was 600 nmol/L lower in the group receiving no FA versus 800 µg FA/day. Importantly, all women remained above serum and RBC folate concentrations indicative of deficiency, >6.8 and >305 nmol/L, respectively (Institute of Medicine, 1998). The folate metabolites at baseline are already high, possibly due to the presence of fortified foods. Our previous study conducted in Australia (Hunt et al., 2020) showed that contemporary levels of RBC folates in women of reproductive age (18–44 y) are 942 (95% CI, 887–1012) nmol/L. These findings are similar to those reported among US women aged 12–49 y recorded during 2007–2010, which showed levels at 995 (95% CI, 972–1020) nmol/L, and during 2011–2016, where levels were 1020 (95% CI, 998–1040) nmol/L (Pfeiffer et al., 2019).

Maternal and neonatal birth outcomes were collected via maternal report for the sole purpose of treatment group comparisons, as the study lacked sufficient power to assess clinical outcomes. Notably, our findings indicated that infants born to mothers who received FA supplementation had greater head circumference at birth than those in the no FA intervention group. However, given the limited number of participants and the absence of multiplicity correction for secondary outcomes, this observation might be attributed to random variation.

Our study has many strengths, including a low attrition rate and a high rate of supplement adherence. Also, UMFA analyses were conducted by the same laboratory in Norway as Pentieva et al., allowing us to compare results. We asked women to refrain from taking their study supplement for 24 h before their blood sample collection to reduce the variation in UMFA caused by recent high‐dose FA exposure. A limitation of our study is the absence of a baseline maternal blood sample at enrollment due to COVID‐19 restrictions, which meant we could not examine changes in UMFA over time.

In conclusion, our trial showed that removing FA from prenatal multivitamin and mineral supplements reduced the serum UMFA concentration at 36 weeks gestation; however, UMFA concentrations were low in both groups. UMFA, even when measured under standardized conditions, has a high within‐subject variation. A nutritional biomarker that is influenced by recent dietary intake should be measured under standardized conditions (i.e., fasting); however, this was not possible given our study design and taking the safety of pregnant women into account (Gibson, 2023). Moreover, there is no cutoff concentration based on clinical outcomes for UMFA, above which there is increased risk of poor maternal and child outcomes (Gibson, 2023). Thus, UMFA may not be the best biomarker for chronic excessive FA ingestion. Our findings do not prove that excessive maternal FA supplementation or UMFA does not cause harm. There is no question that FA supplementation is essential before and in early pregnancy, but investigating excess intake, especially in countries with mandatory fortification, is warranted. High‐quality randomized controlled trials powered with clinical endpoints are needed to resolve concerns regarding the potential adverse effects of excessive FA intakes in late pregnancy on maternal and child health.

## AUTHOR CONTRIBUTIONS

Karen P. Best, Timothy J. Green, Dian C. Sulistyoningrum, Maria Makrides, Debra J. Palmer, Monika Skubisz and Simon Wood conceived the trial, proposed the trial design and the supplement formulation; Karen P. Best, Dian C. Sulistyoningrum, Timothy J. Green and Monika Skubisz conducted the trial; Adrian McCann, Per Magne Ueland and Dian C. Sulistyoningrum analyzed the blood samples; Thomas R. Sullivan advised on sample size, developed the statistical analysis plan and conducted the statistical analyses; Dian C. Sulistyoningrum, Timothy J. Green and Karen P. Best drafted the manuscript; all authors provided critical input into the manuscript and approved the submitted version.

## CONFLICT OF INTEREST STATEMENT

Simon Wood is a consultant for the Factors Group of Companies. The remaining authors declare no conflict of interest.

## Supporting information

Supporting information.

## Data Availability

The data that support the findings of this study are available on request from the corresponding author. The data are not publicly available due to privacy or ethical restrictions.
