# Supplementary material for: Maternal serum unmetabolized folic acid concentration following multivitamin and mineral supplementation with or without folic acid after 12 weeks gestation: A randomized controlled trial
Source: Matern Child Nutr. 2024 May 23;20(4):e13668. doi: 10.1111/mcn.13668 (PMC11574634; doi:10.1111/mcn.13668)
Supplement: Supplementary file 1 — Supporting information. [file MCN-20-e13668-s001.docx]

**Supporting information**

This appendix has been provided by the authors to give readers additional information about their work.

Supplement to: Sulistyoningrum DC, Sullivan TR, Skubisz M, Palmer DJ, Wood S, Snel MF, Trim PJ, Makrides M, Green TJ, Best KP. **Detection of maternal serum unmetabolized folic acid following multivitamin and mineral supplementation with or without folic acid after 12 weeks’ gestation: a randomized controlled trial.**

**Table 1. Ingredients of Supplements for Intervention (no folic acid) and Control (800 µg folic acid) Groups**

| Ingredients | No folic acid | 800 µg folic acid | unit |
| --- | --- | --- | --- |
| folic acid | 0 | 0.8 | mg |
| calcium | 250 | 250 | mg |
| Iron | 27 | 27 | mg |
| thiamine | 1.4 | 1.4 | mg |
| riboflavin | 1.4 | 1.4 | mg |
| niacinamide | 18 | 18 | mg |
| vitamin B-6 | 1.9 | 1.9 | mg |
| vitamin B-12 | 2.6 | 2.6 | mcg |
| pantothenic acid | 6 | 6 | mg |
| biotin | 30 | 30 | mg |
| vitamin C | 85 | 85 | mg |
| vitamin E | 13.5 | 13.5 | IU |
| magnesium | 50 | 50 | mg |
| zinc | 7.5 | 7.5 | mg |
| manganese | 2.0 | 2.0 | mg |
| iodine | 0.22 | 0.22 | mg |
| copper | 1 | 1 | mg |
| selenium | 30 | 30 | mcg |
| Vitamin D3 | 10 | 10 | mcg |
| b-carotene | 2500 | 2500 | IU |

**Figure 1. Histograms representing frequencies of serum unmetabolized folic acid, serum folate and red blood cell folate concentrations**


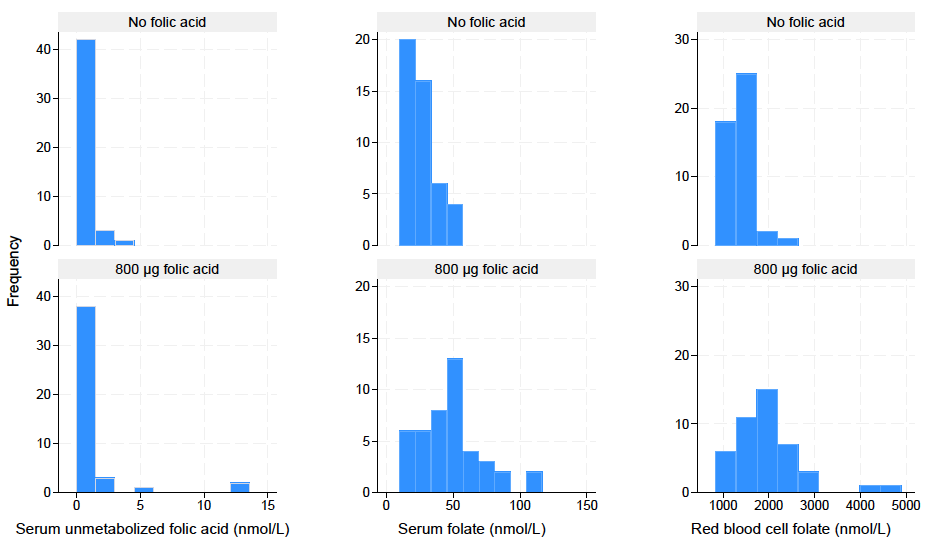


**Table 2. Adverse events**

|  | No FA | 800 µg FA | Fisher exact test p-value |
| --- | --- | --- | --- |
| **1-week post-randomization** | | | |
| Diarrhea | 2/50 (4%) | 3/49 (6%) | 0.68 |
| Nausea | 13/51 (25%) | 14/49 (29%) | 0.82 |
| Vomiting | 7/51 (14%) | 6/49 (12%) | >0.99 |
| Eructation | 3/51 (6%) | 5/48 (10%) | 0.48 |
| Constipation | 6/51 (12%) | 7/49 (14%) | 0.77 |
| Other symptom | 2/51 (4%) | 2/49 (4%) | 1.00 |
| **20 weeks gestation** | | | |
| Diarrhea | 4/47 (9%) | 6/46 (13%) | 0.52 |
| Nausea | 13/47 (28%) | 14/46 (30%) | 0.82 |
| Vomiting | 7/47 (15%) | 6/46 (13%) | >0.99 |
| Eructation | 10/47 (21%) | 11/46 (24%) | 0.81 |
| Constipation | 15/47 (32%) | 19/46 (41%) | 0.39 |
| Other symptom | 1/47 (2%) | 6/46 (13%) | 0.06 |
| **24 weeks gestation** | | | |
| Diarrhea | 2/45 (4%) | 3/43 (7%) | 0.67 |
| Nausea | 4/45 (9%) | 10/43 (23%) | 0.08 |
| Vomiting | 1/45 (2%) | 4/43 (9%) | 0.20 |
| Eructation | 7/45 (16%) | 11/43 (26%) | 0.30 |
| Constipation | 11/45 (24%) | 12/43 (28%) | 0.81 |
| Other symptom | 2/45 (4%) | 2/43 (5%) | >0.99 |
| **28 weeks gestation** | | | |
| Diarrhea | 4/46 (9%) | 7/43 (16%) | 0.34 |
| Nausea | 3/46 (7%) | 5/43 (12%) | 0.48 |
| Vomiting | 0/46 (0%) | 1/43 (2%) | 0.48 |
| Eructation | 6/46 (13%) | 8/43 (19%) | 0.57 |
| Constipation | 8/46 (17%) | 16/43 (37%) | 0.06 |
| Other symptom | 1/46 (2%) | 3/43 (7%) | 0.35 |
| **32 weeks gestation** | | | |
| Diarrhea | 4/46 (9%) | 3/44 (7%) | >0.99 |
| Nausea | 2/46 (4%) | 7/44 (16%) | 0.09 |
| Vomiting | 1/46 (2%) | 2/44 (5%) | 0.61 |
| Eructation | 4/46 (9%) | 8/44 (18%) | 0.23 |
| Constipation | 7/46 (15%) | 9/44 (20%) | 0.59 |
| Other symptom | 1/46 (2%) | 2/43 (5%) | 0.61 |
| **36 weeks gestation** | | | |
| Diarrhea | 3/45 (7%) | 4/43 (9%) | 0.71 |
| Nausea | 5/45 (11%) | 6/43 (14%) | 0.76 |
| Vomiting | 1/45 (2%) | 2/43 (5%) | 0.61 |
| Eructation | 2/45 (4%) | 5/43 (12%) | 0.26 |
| Constipation | 6/45 (13%) | 7/43 (16%) | 0.77 |
| Other symptom | 4/45 (9%) | 2/43 (5%) | 0.68 |
